# How Do Childhood Diagnoses of Type 1 Diabetes Cluster in Time?

**DOI:** 10.1371/journal.pone.0060489

**Published:** 2013-04-03

**Authors:** Colin R. Muirhead, Timothy D. Cheetham, Simon Court, Michael Begon, Richard J. Q. McNally

**Affiliations:** 1 Institute of Health and Society, Newcastle University, Newcastle upon Tyne, United Kingdom; 2 Institute of Genetic Medicine, Newcastle University, Newcastle upon Tyne, United Kingdom; 3 School of Biological Sciences, University of Liverpool, Liverpool, United Kingdom; Mayo Clinic, United States of America

## Abstract

**Background:**

Previous studies have indicated that type 1 diabetes may have an infectious origin. The presence of temporal clustering—an irregular temporal distribution of cases—would provide additional evidence that occurrence may be linked with an agent that displays epidemicity. We tested for the presence and form of temporal clustering using population-based data from northeast England.

**Materials and Methods:**

The study analysed data on children aged 0–14 years diagnosed with type 1 diabetes during the period 1990–2007 and resident in a defined geographical region of northeast England (Northumberland, Newcastle upon Tyne, and North Tyneside). Tests for temporal clustering by time of diagnosis were applied using a modified version of the Potthoff-Whittinghill method.

**Results:**

The study analysed 468 cases of children diagnosed with type 1 diabetes. There was highly statistically significant evidence of temporal clustering over periods of a few months and over longer time intervals (*p*<0.001). The clustering within years did not show a consistent seasonal pattern.

**Conclusions:**

The study adds to the growing body of literature that supports the involvement of infectious agents in the aetiology of type 1 diabetes in children. Specifically it suggests that the precipitating agent or agents involved might be an infection that occurs in “mini-epidemics”.

## Introduction

Both genetic and environmental factors are implicated in the aetiology of type 1 diabetes [1]. The involvement of environmental factors is suggested because incidence has exhibited general temporal increases and does not display geographical uniformity [2]–[7]. Furthermore, increased incidence has been reported in areas of less dense population or less deprivation [8]–[12]. These and other findings suggest that infectious agents might be involved in aetiology [13]–[16].

An earlier analysis of population-based data on type 1 diabetes in children in northeast England found marginally significant evidence of space-time clustering [17]. This evidence was strongest for females and for cases in more densely populated areas. A subsequent analysis of annual incidence rates in the same region demonstrated a 6-year cyclical pattern, suggesting the possible role of an infectious agent in the aetiology of this disease [18].

The present study is concerned with the detection of irregular temporal distributions of cases of type 1 diabetes in children. A general irregular temporal distribution of cases that is not confined to one particular time period is known as ‘temporal clustering’. This sort of clustering could arise because there are a small number of time periods with greatly increased incidence or a large number of time periods with moderately increased incidence. Temporal clustering might provide further evidence that the disease has an infectious or local environmental component. This irregular pattern contrasts with seasonal effects that occur at the same time each year and necessitates different statistical methods from those used to identify seasonality. We also sought to minimise the potential for confounding arising from population movements or population heterogeneity. We therefore used novel methodology to analyse data from an area with a stable population, with low levels of inward or outward migration, and whose population is ethnically homogeneous [19]–[21]. As a positive control, we also applied this methodology to data for a known infectious disease, namely influenza.

## Materials and Methods

### Patients

The proposal for this work was reviewed by the institutional R&D department. This department decided that ethical review was not required, as the patient data used were deemed to be non-identifiable.

The study analysed data for children aged 0–14 years who were diagnosed with type 1 diabetes between 1 January 1990 and 31 December 2007 and resident in a region of northeast England, namely Northumberland, Newcastle upon Tyne and North Tyneside. In order to help to ensure a high level of case ascertainment, cases were identified from several independent sources, as follows:

1. paediatric and adult clinic databases;
2. diabetes team admission diaries;
3. in/out patient records;
4. a regional adult diabetes register.

Subjects considered to be at high risk of developing type 1 diabetes (such as first degree relatives) were not actively screened in our locality in the absence of intervention studies or treatment options at the time. Hence presentation was not by any other means.

There were originally three paediatric services in the study region managing young patients with diabetes up to the age of 16 to 18 years, although two of these services merged in 2001. In our subjects, the diagnosis of type 1 diabetes was made by the managing paediatricians (all of whom had a major interest in diabetes) on the basis of a blood glucose concentration that was greater than 11.1 mmol/l in association with a classical history of polyuria, polydipsia and weight loss. The number of cases of non-type 1 diabetes in our region is very small. To be certain about this, we reviewed the clinic population from one of the two services in the locality and the number of established cases of non-type 1 diabetes (type 2 diabetes or patients with single gene defects such as HNF1alpha or HNF1beta) managed within the service during the period of the study was less than 2%. Patients with established type 2 diabetes were not included in the analysis. We also conducted cross-checks to be certain that the management of childhood cases in primary care was not common practice; these checks identified only one instance where a young person aged less than 15 years was not referred promptly to regional paediatric services. Paediatricians in the surrounding area (including Cumbria and Southern Scotland) were also contacted so that we could be certain that patients born in the study region were not being managed in clinics elsewhere.

When case identification was undertaken and the database compiled, a date of 1^st^ July was entered if cross-referencing demonstrated the year of diagnosis but there was uncertainty about the specific date of diagnosis. Inspection of the data at the time of the current analysis indicated that 58 out of 526 cases, mostly diagnosed in the 1990s, had been assigned to 1^st^ July. These 58 cases were excluded from analyses of variation within years. However, because the year of diagnosis for these cases was correct, they were retained in the analysis between years.

An earlier analysis of space-time clustering in this study region [17] had slightly fewer cases than that used here for the analyses within years (i.e. 457 vs. 468). This is because the earlier analysis was restricted to cases with accurate geo-referencing. However, this restriction is not necessary here, because we are examining temporal clustering and are confident that all of the cases arose within the study region.

### Statistical methods

The analysis was undertaken using the approach described by Muirhead [22] and by Muirhead and Butland [23] to look for disease clustering, but applied here to temporal rather than spatial clustering. In brief, this involved a test derived by Potthoff and Whittinghill [24], [25] to look for extra-Poisson variation, assuming that the numbers of cases in the study units (taken here to represent time periods) are distributed as negative binomial with the ratio of the variance to the mean equal to a constant, namely 1+β. If β equals 0, then the observations are distributed as Poisson, whereas if β is greater than 0, then the observations exhibit extra-Poisson variation, i.e. are over-dispersed relative to Poisson. Having conditioned on the total number of cases, the Potthoff-Whittinghill (P-W) statistic can be recast as:

*Σ (number of pairs of cases in the time period/expected number of cases in the time period)*

where the sum is over all the time periods under study. By focusing on the numbers of pairs of cases, this statistic magnifies the impact of those time periods during which larger numbers of cases are observed than would be expected under Poisson variation; in other words, those periods during which clustering of cases arises. The standardised version of the P-W statistic used here [23] provides an estimate of β, i.e. the degree of extra-Poisson variation, and is the most powerful test to detect small values of β [24], [25].

Muirhead [22] and Muirhead and Butland [23] proposed an extension of this approach to study a hierarchy of geographical areas. This has been implemented here but based on time periods rather than geographical areas, the aim being to distinguish between short-term and longer-term effects. For example, having conditioned on the total number of cases observed in each calendar year, a version of the P-W statistic can be calculated using the numbers of cases in each month and summing contributions to the P-W statistic over years. In this way, an estimate can be obtained of the extra-Poisson variation between months within years; in other words, removing effects that might occur over periods of years or decades when trying to identify effects arising over months. In addition, data aggregated over quarters of a year (defined as January to March; April to June; July to September; and October to December) and over fortnights (defined pragmatically as the first 15 days of the calendar month, or the first 14 days for February, versus the remainder of the month) have been analysed, so as to look (for example) for effects between fortnights within months, between months within quarters, and between quarters within years. Periods shorter than a fortnight were not considered, so as to minimise the possible impact of referral patterns (e.g. differences between weekends and weekdays, as reported by Mooney et al [26]).

Expected numbers of cases were calculated using annual population sizes for the study region, standardised by sex and age at diagnosis. For periods of equal length within any calendar year, the expected number of cases was assumed to be constant.

Statistical significance was assessed by conducting 10000 simulations of the standardised P-W statistic under the assumption of Poisson variation, i.e. β equal to 0. Particular attention was directed to p-values less than 0.05. Since the aim was to test for over-dispersion (i.e. β>0, reflecting a tendency for cases to aggregate) rather than under-dispersion (β<0), one-sided tests were used. Diagnostics described by Muirhead [22] were used to see if particular time periods had a strong influence on the evidence for extra-Poisson variation. Sub-group analyses were conducted using data split by age (0–4, 5–9 and 10–14 years) and sex. Data were not availability on ethnicity. However, over 98% of people living in the study region were known to be white British [21].

The statistical approach used here contrasts with that employed in analyses of seasonality in type 1 diabetes (e.g. [27], [28]). These studies involved fitting models that allowed for peak and troughs in rates, possibly with a long-term linear trend (e.g. as in [29], [30]). However, these models did not allow for variation between years in peaks or troughs, whereas the approach taken here does not assume that a similar pattern would arise each year.

### Check on methodology

As a positive control, the P-W methodology was also applied to data on laboratory-confirmed influenza hospitalisations at ages 0–4 years, based on locations in California that participate in the United States Centers for Disease Control and Prevention's Emerging Infections Programs (http://www.cdc.gov/ncezid/dpei/eip/). Data on the numbers of cases per week, for each flu season from 2006–07 through to 2011–12, were obtained via http://gis.cdc.gov/GRASP/Fluview/FluHospRates.html. These data covered weeks during the final quarter of each calendar year and the first quarter of the following year, as well as additional weeks either side of each quarter for some flu seasons. The calculation of expected numbers of cases took account of the number of weeks in each quarter and flu season. A total of 370 cases arose during the study period.

## Results

### Influenza

**Figure 1:**
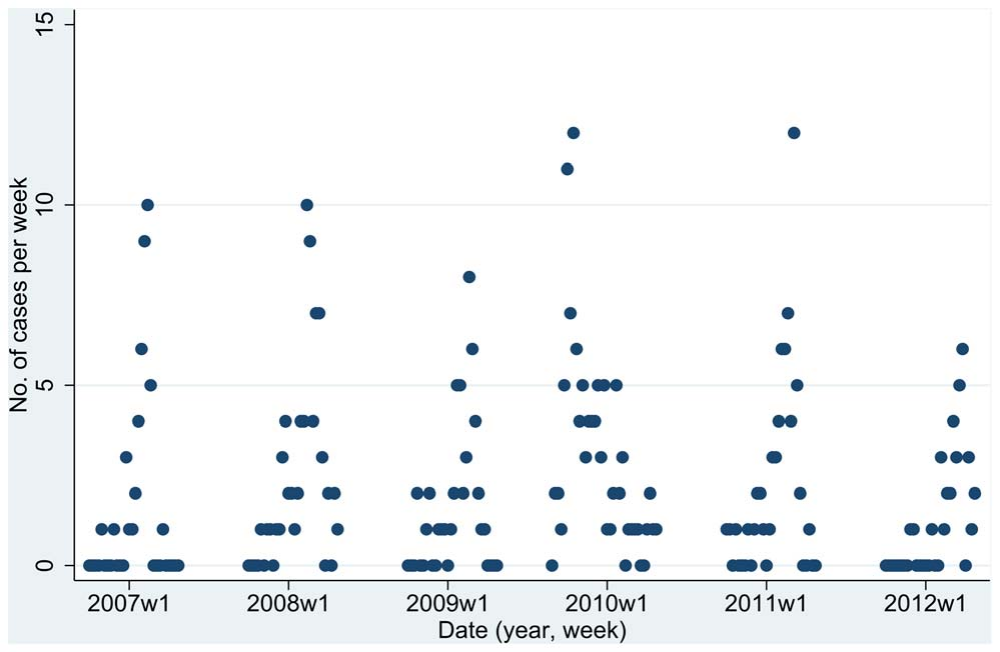
Weekly influenza hospitalisations by calendar period (California, 0–4 years).

Figure 1 shows the weekly numbers of influenza hospitalisations in locations in California and Table 1 shows the results from applying the P-W method to these data. There was highly significant extra-Poisson variation in the numbers of cases not only between flu seasons and between quarters within flu seasons, but also between months within quarters (p<0.001 in each instance). This reflects the patterns evident from Figure 1. In contrast, there was no significant extra-Poisson variation between weeks within months (p = 0.13; see top-left corner of Table 1). Thus the variation in weekly numbers of cases mainly reflects effects at the level of months and longer periods.

**Table 1:** Application of the Potthoff-Whittinghill technique to detecting temporal clustering of influenza hospitalisations among children aged 0–4 years in California^a^ during the flu seasons from 2006–07 to 2011–12 inclusive.

PW^b^ (SE)^c^				
*one-sided p-value^d^*				
Type of analysis	Within months	Within quarters	Within flu seasons	Within full study period
Between weeks	0.170 (0.149)	1.22 (0.11)	2.25 (0.11)	2.29 (0.10)
	*p = 0.13*	*p<0.001*	*p<0.001*	*p<0.001*
Between months		5.83 (0.27)	9.94 (0.23)	9.21 (0.22)
		*p<0.001*	*p<0.001*	*p<0.001*
Between quarters			20.21 (0.58)	14.45 (0.43)
			*p<0.001*	*p<0.001*
Between flu seasons				6.79 (0.63)
				*p<0.001*

Notes:

Based on locations participating in the California Emerging Infections Program.

a PW is the one-step estimate of β, the extra-Poisson variation, calculated as S/i(0) in the notation of Muirhead [22].

b SE is the standard error of PW in the absence of extra-Poisson variation, calculated as 1/√i(0) in the notation of Muirhead [22].

c p-values have been calculated using 10000 simulations of PW, assuming Poisson variation. All p-values are one-sided.

### Type 1 diabetes

**Figure 2:**
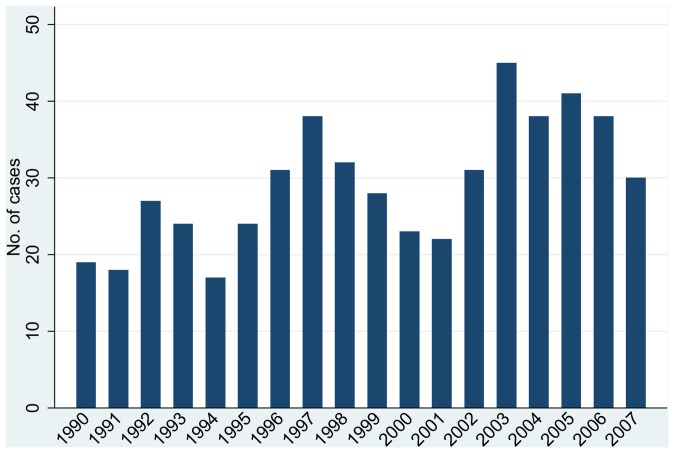
Annual number of cases of type 1 diabetes at ages 0–14 years in Northumberland, Newcastle upon Tyne and North Tyneside by year of diagnosis.

Figure 2 shows the annual number of type 1 diabetes cases within the study region in northeast England by year of diagnosis. A clear long-term cyclical pattern is evident, in line with that reported by McNally et al [18]. The P-W analysis found strong evidence of extra-Poisson variation in the numbers of cases between years (p<0.001; see the bottom right-hand corner of Table 2). No one year had a strong influence on these results. A sensitivity analysis that excluded diagnoses of 1^st^ July gave a higher estimate of extra-Poisson variation between years (β of 3.666 rather than 1.936), possibly reflecting the greater proportion of such diagnoses in the early part of the study period. Sub-group analyses found evidence for extra-Poisson variation between years (p<0.05) separately for females and males, and at ages 0–4 and 10–14 years, but not at ages 5–9 years.

**Table 2:** Application of the Potthoff-Whittinghill technique to detecting temporal clustering of diagnoses of type 1 diabetes at ages 0–14 years in Northumberland, Newcastle upon Tyne and North Tyneside during 1990–2007 inclusive.

		PW^a^ (SE)^b^		
		*one-sided p-value* c		
Type of analysis d	Within months	Within quarters	Within years	Within full study period
Between fortnights	−0.310 (0.157)	−0.110 (0.086)	−0.004 (0.071)	0.132 (0.068)
	*p = 0.98*	*p = 0.90*	*p = 0.51*	*p = 0.032*
Between months		0.010 (0.136)	0.189 (0.103)	0.459 (0.097)
		*p = 0.46*	*p = 0.039*	*p = 0.001*
Between quarters			0.707 (0.197)	1.530 (0.168)
			*p<0.001*	*p<0.001*
Between years				1.936 (0.343)
				*p<0.001*

Notes:

a PW is the one-step estimate of β, the extra-Poisson variation, calculated as S/i(0) in the notation of Muirhead [22].

b SE is the standard error of PW in the absence of extra-Poisson variation, calculated as 1/√i(0) in the notation of Muirhead [22].

c p-values have been calculated using 10000 simulations of PW, assuming Poisson variation. All p-values are one-sided.

d The analysis between years was based on all 526 cases, whereas the other analyses excluded the 58 cases with a diagnosis date of 1^st^ July.

**Figure 3:**
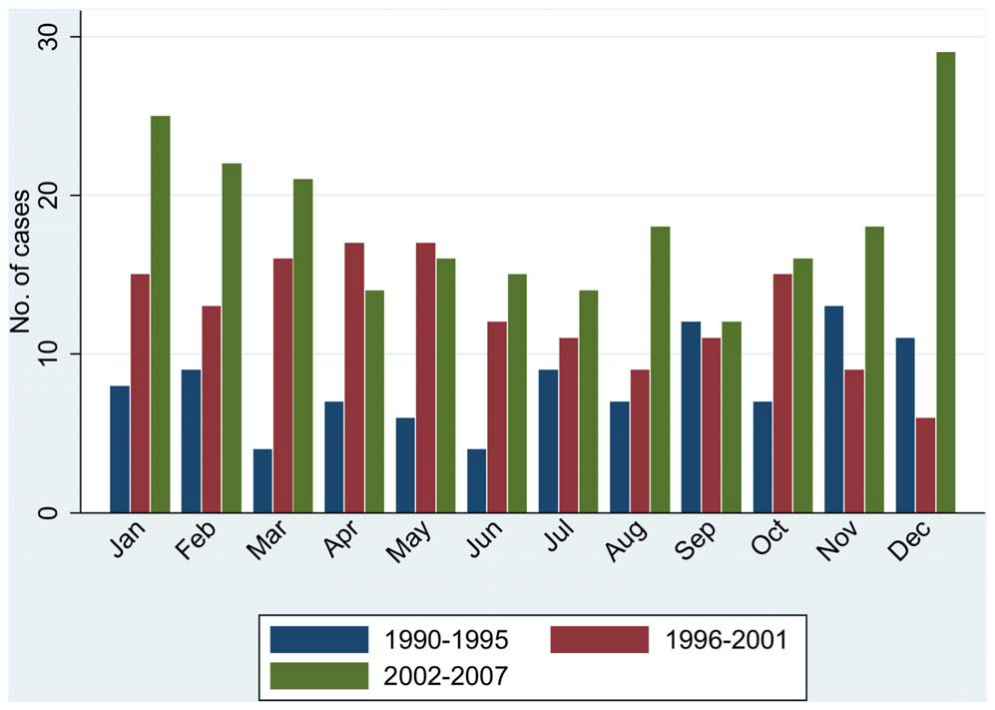
Number of cases of type 1 diabetes at ages 0–14 years in Northumberland, Newcastle upon Tyne and North Tyneside by calendar month and period of diagnosis.

Figure 3 shows the total number of cases observed during each calendar month, separately for consecutive six-year periods (1990–1995, 1996–2001 and 2002–2007) so as to reduce the impact of long-term variation; diagnosis dates of 1^st^ July have been excluded. This Figure not only indicates variation within years, but that the form of this variation changed during the study period. The P-W analysis suggests that this variation within years occurs principally between quarters of a year. In particular, there was statistically significant evidence of extra-Poisson variation in the numbers of cases between quarters within years (*p*<0.001; see penultimate entry on the diagonal in Table 2); no one quarter had a strong influence on this result. The evidence for extra-Poisson variation between quarters within years was most marked for females and at ages 5–9 and 10–14 years. In contrast, there was no significant evidence of extra-Poisson variation between months within quarters, nor between fortnights within months, either overall (Table 2) or in sub-group analyses (results not shown). Indeed, there was some evidence of under-dispersion in the numbers of cases between fortnights within months. The analysis between months within years, as well as the analyses between fortnights within either years or the full study period, also showed significant extra-Poisson variation (p<0.05; Table 2), probably reflecting the impact of variation both between quarters and between years.

## Discussion

This analysis has not only confirmed the long-term cyclical variation of childhood type 1 diabetes reported previously [18], but has also established effects over shorter periods. Having allowed for annual differences, the numbers of cases in each quarter of a year showed greater variation than would be expected under the Poisson distribution. In contrast, there was no evidence of extra-Poisson variation within quarters of a year. These results indicate that temporal clustering of cases occurs over periods of a few months, in addition to cyclical variation over years. This pattern of occurrence is consistent with the involvement of exogenous agents, such as an infection, that may exhibit epidemicity. We also checked the methodology using data for influenza in children, based on a similar total number of cases as for the diabetes dataset and again with confirmed diagnoses. The P-W method detected temporal clustering of influenza at levels of months, quarters of a year and flu seasons. Consequently, the methodology used here should have good power to detect clinically meaningful clustering.

Several studies, e.g. [5], [27], [31], have reported seasonality in dates of diagnosis of childhood type 1 diabetes, often with peaks in October to January and troughs in June to August for centres in the northern hemisphere. However, not all studies have reported the same pattern; indeed, a recent analysis from Denmark [28] suggested that the seasonal pattern might change over time. The present study adds to the evidence for such an irregular temporal pattern (i.e. temporal clustering). The statistical method used here is well-placed to identify changing patterns of the form shown in Figure 3 because-unlike the usual analyses of seasonality-it does not assume that peaks or troughs occur at the same time each year.

Genetic factors, notably the HLA system, influence susceptibility to type 1 diabetes; however, the increases in disease incidence seen in many countries in recent years [2]–[5], [7] cannot be explained by genetic factors alone and highlight the role of the environment in disease evolution. Factors such as the increase in population obesity and associated insulin resistance have been viewed by some as a plausible explanation for the increase in diabetes incidence in young people, but cannot easily be reconciled with the present finding of temporal clustering [32]. The finding of such an irregular pattern in incidence is consistent with the involvement of an infectious agent which itself displays an irregular pattern in the environment. Evidence for a role of infections in the aetiology of type 1 diabetes comes from epidemiological studies of, for example, birth order [33], interbirth interval [34], rural locality [11], population mixing [35], day care attendance [36] and recorded neonatal illnesses [37], whereas studies of recorded infections in early life have been inconsistent [38]–[41]. Experimental evidence also supports the role of an infectious agent or agents in the development of type 1 diabetes [14], [15],[42] and the involvement of infections in northeast England is supported by the previous identification of space-time clustering in our locality [17]. The evidence from the present study for temporal clustering over periods of a few months suggests that an infectious agent or agent may act as a final trigger in the development of the disease amongst susceptible individuals.

A number of candidate viruses have been implicated in the aetiology of type 1 diabetes including enteroviruses, rotavirus, mumps, cytomegalovirus, rubella and Ljungan virus [13],[43]–[45]. For example, a recent systematic review found a strong association between enterovirus infection and both type 1 diabetes-related autoimmunity and clinical type 1 diabetes [46]. The type of clustering found in the present study suggests that the underlying pattern in the risk of such an agent being passed from a reservoir host may exhibit a natural “epidemicity”. The most common type of reservoir for zoonotic infections, especially in temperate regions, is wild rodent populations [47]. Patterns of variation in the risk of transmission from them will reflect a combination of variation in infection prevalence and in host abundance. Wild rodents in the north of England are known to exhibit multi-annual cycles of abundance, though these typically have a period of 3–4 rather than 6 years [48], but it is also known that superimposed upon host abundance patterns, infection prevalence and the timing of peaks of prevalence may vary markedly from year to year, as a result of interplay between prevailing weather, host demography and infection dynamics [49], [50]. However, making a direct and detailed link between the intra- and multi-annual patterns observed here, and intra- and multi-annual patterns in the infection dynamics of wild rodents that are a potential natural reservoir, will require further research focused specifically on elaborating those dynamics.

The precise mechanism whereby an organism such as an enterovirus might affect the evolution of type 1 diabetes is unclear [51], although islet cell damage could be a key part of this process (reviewed in [13]). The level of exposure could affect the immune system in a manner that is dependent on factors such as age and genotype. The hygiene hypothesis [52], [53] has been proposed as a potential explanation for the association between greater disease incidence and improved sanitation. This trend has been observed both between and within countries [8]–[10],[54]. High levels of exposure to infectious agents in the population as a whole may refine immune responses, with the interaction between micro-organism and individual potentially decreasing as well as increasing the likelihood of disease development [55]. In particular, discussion of the hygiene hypothesis in relation to autoimmune diseases has highlighted the key role of timing of exposure, in that certain viruses might provoke autoimmunity when given late but be protective when given very early [53]. Laboratory studies of type 1 diabetes provide support for this [56], [57], whereas epidemiological studies of type 1 diabetes and recorded infections in the first year of life are inconsistent [38]–[40]. Nevertheless, the lack of evidence for temporal clustering between quarters within years at ages under 5 years in our study might be explained by a protective effect of exposure to infectious agents at very young ages. Furthermore, the time between exposure to virus infections and disease onset is likely to vary between individuals and may be shorter amongst susceptible individuals (i.e. those exposed at an inappropriate stage of maturation). Thus, our findings of temporal clustering at older ages are consistent with virus infection or infections acting on the immune system of susceptible individuals and leading to clinically observable disease in some of these individuals shortly thereafter.

Suboptimal ascertainment with incomplete data collection and the potential for patients from one locality to be managed in centres outside the area studied are important considerations in a study such as this. We collected and cross-checked data from sources other than local paediatric clinic databases and also liaised with neighbouring regions to make sure that these factors would not be significant confounders in our analyses and that ascertainment was high. We remain unable to reconcile highly significant temporal clustering with aberrations in the way data have been collected or with cases of diabetes in young people being classified incorrectly.

In conclusion, the present study adds substantively to the growing body of literature that supports the involvement of infectious agents in the aetiology of type 1 diabetes in children. Specifically it suggests that the precipitating agent or agents involved might be an infection that occurs in “mini-epidemics.”
